# Evolution and Development of the Inner Ear Efferent System: Transforming a Motor Neuron Population to Connect to the Most Unusual Motor Protein via Ancient Nicotinic Receptors

**DOI:** 10.3389/fncel.2017.00114

**Published:** 2017-04-24

**Authors:** Bernd Fritzsch, Karen L. Elliott

**Affiliations:** Department of Biology, University of IowaIowa, IA, USA

**Keywords:** ear, development, evolution, efferents, facial branchial motor neurons, nicotinic receptors, alpha 9, alpha 10

## Abstract

All craniate chordates have inner ears with hair cells that receive input from the brain by cholinergic centrifugal fibers, the so-called inner ear efferents (IEEs). Comparative data suggest that IEEs derive from facial branchial motor (FBM) neurons that project to the inner ear instead of facial muscles. Developmental data showed that IEEs develop adjacent to FBMs and segregation from IEEs might depend on few transcription factors uniquely associated with IEEs. Like other cholinergic terminals in the peripheral nervous system (PNS), efferent terminals signal on hair cells through nicotinic acetylcholine channels, likely composed out of alpha 9 and alpha 10 units (Chrna9, Chrna10). Consistent with the evolutionary ancestry of IEEs is the even more conserved ancestry of Chrna9 and 10. The evolutionary appearance of IEEs may reflect access of FBMs to a novel target, possibly related to displacement or loss of mesoderm-derived muscle fibers by the ectoderm-derived ear vesicle. Experimental transplantations mimicking this possible aspect of ear evolution showed that different motor neurons of the spinal cord or brainstem form cholinergic synapses on hair cells when ears replace somites or eyes. Transplantation provides experimental evidence in support of the evolutionary switch of FBM neurons to become IEEs. Mammals uniquely evolved a prestin related motor system to cause shape changes in outer hair cells regulated by the IEEs. In summary, an ancient motor neuron population drives in craniates via signaling through highly conserved Chrna receptors a uniquely derived cellular contractility system that is essential for hearing in mammals.

## Introduction

The central nervous system (CNS) of craniate chordates connects to peripheral target organs via two sets of nerve fibers comprising the peripheral nervous system (PNS):

1. Afferent (centripetal) fibers bring information into the CNS. Afferents develop from neural crest or placode-derived sensory neurons of spinal and cranial ganglia (Northcutt, 2005). Central axons end in the alar plate of the spinal cord and hindbrain whereas the distal processes contact skin and muscle associated receptors (neural crest ganglia) and inner ear, lateral line (if present), and taste bud sensory cells (placode ganglia). All ganglion neurons are glutamatergic with variable additional transmitters and require the bHLH genes Neurog1 and 2 for their development (Ma et al., 2000).
2. Efferent (centrifugal) fibers bring information to the PNS. Efferents develop in vertebrates from basal plate-derived motor neurons and axons project either through ventral roots (somatic and visceral motor neurons of the spinal cord), or dorsal roots (only branchial and visceral motor neurons of the hindbrain). Efferent fibers reach striated muscle fibers or neural crest-derived visceral ganglia of the autonomic system. Efferents use acetylcholine as the primary transmitter with variable additional transmitters. Efferents, branchial, visceral, and most ocular cranial motor neurons require Phox2a and/or 2b for their normal development (Tiveron et al., 2003) whereas spinal somatic and visceral motor neurons require the bHLH gene Olig2 (Espinosa-Medina et al., 2016).
3. Among the special cranial senses, the inner ear and the eye are unique in that they receive efferent (centrifugal) innervation. The efferent innervation of the craniate retina is highly variable and appears to reflect the original connection of the diencephalic brain that evolved into the vertebrate retina (Manns and Fritzsch, 1991; Ward et al., 1991; Repérant et al., 2007; Lamb, 2013). In contrast to the visual system, the highly divergent original findings on the inner ear efferent (IEE) system (Fritzsch et al., 2016a) soon concentrated on a single theme: efferents to the ear being evolutionary derived from facial branchial motor neurons (FBMs) (Roberts and Meredith, 1992; Sienknecht et al., 2014). Further work demonstrated that IEEs diverge through selective expression of certain transcription factors from FBMs to end on inner ear sensory hair cells (Karis et al., 2001; Sienknecht et al., 2014).

This review will provide evidence for the evolutionary and developmental reorganization of FBMs to IEEs that terminate on hair cells carrying an ancient nicotinic acetylcholine receptor (Elgoyhen et al., 1994; Lustig et al., 2001).

## Evolution of the vertebrate ear and efferent system to hair cells in craniate vertebrates

**Figure 1:**
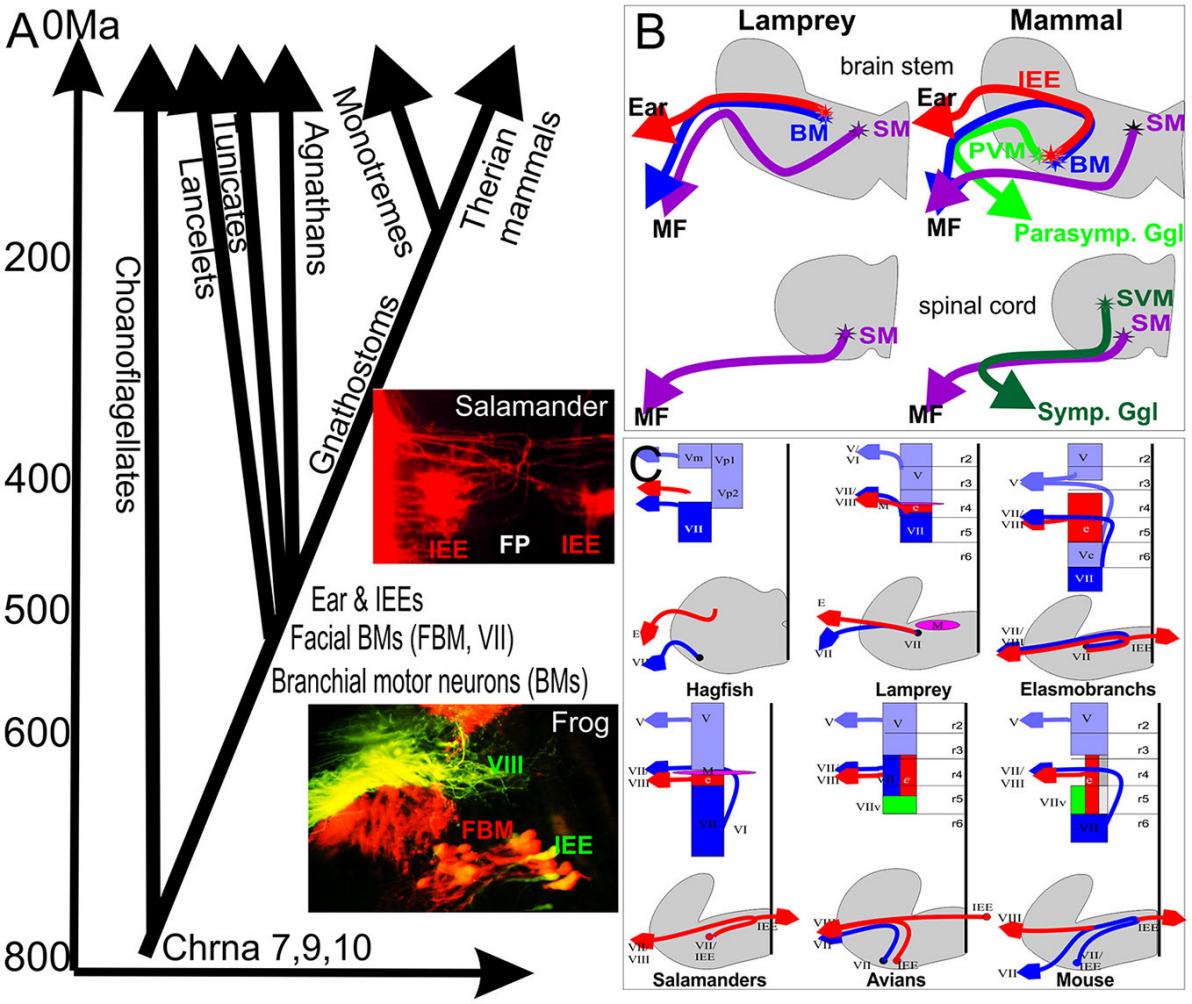
**(A)** The evolutionary context of vertebrate inner ear evolution, motor neuron, and nicotinic acetylcholine receptors is depicted against the molecularly defined history of major events in animal history. Evolution of nicotinic subunits associated with the vertebrate inner ear (9,10) coincides with the split of the single celled ancestor of animals from the single celled outgroup, the choanoflagellates (around 800 million years). Evolution of chordates (540 million years ago) coincides with the evolution of branchial motor neurons whereas evolution of an ear and inner ear efferents (IEEs) overlaps with the evolution of craniate IEEs (~520 million years ago). Inserts show overlap of IEEs (green, VIII) and FBMs (red) in frogs and bilateral distribution of IEEs with branching fibers in the flor plate (FP) in a salamander. **(B)** Jawless craniates like Lampreys apparently have only somatic motor neurons (SM) in the spinal cord and branchial motor neurons (BM) in the brain partially overlapping with IEEs. Mammals evolved in addition sympathetic visceral motor neurons (SVMs) in the spinal cord and parasympathic visceral motor neurons (PVMs) in the brainstem. **(C)** Laterality and cell and fiber distribution is shown for several vertebrates to emphasize that IEEs are unilateral in hagfish and lamprey (agnathans) but are bilateral with variable segregation of FBMs from IEEs through differential migration in most gnathostomes. Modified after (Fritzsch and Northcutt, 1993; Fritzsch, 1998b, 1999).

Craniates evolved out of chordates some 540 million years ago (Mallatt and Holland, 2013), and jawless and jawed vertebrates separated around 520 million years ago (Figure 1). Craniate ancestors evolved all major cranial sensory organs (eye, ear, nose, taste) from molecular and cellular precursors found in acraniate chordates. In contrast to craniate sensory organs, sensory hair cell precursors of the ear can be traced to chordates and possibly even to the unicellular ancestor of all animals living some 800 million years ago (Fritzsch et al., 2007; Burighel et al., 2011). These sensory cells aggregate to form the vertebrate ear (and lateral line, if present) found only in craniates. The ear is topographically closely associated with the facial nerve and FBMs may be among the oldest branchial motor neurons shared among chordates (Fritzsch and Northcutt, 1993; Dufour et al., 2006). IEEs evolved only with the formation of the craniate ear (Fritzsch, 1999) and are found in agnathans such as lamprey (Fritzsch et al., 1989; Fritzsch, 1998a) and hagfish (Jørgensen et al., 1998) and all jawed vertebrates (Roberts and Meredith, 1992; Fritzsch, 1999). Efferent, vesicle filled terminals to craniate hair cell precursors exist in tunicate chordates (Burighel et al., 2011). Whether the terminals are cholinergic and are branches of FBM axons is unknown. It is also unclear if this is an independently derived feature of tunicates or the primitive condition for chordates. Combined, these data support that IEEs may have co-evolved with the craniate ear.

The very discovery of IEEs was possible due the cholinergic nature of these fibers, a feature shared with motor neurons (Roberts and Meredith, 1992). Despite this compelling evidence, the puzzling array of widely distributed cells traceable from the ear in different vertebrates provided little evidence of a uniform nature of these cells. Major issues associated with the recognition that IEEs are evolutionary-derived FBMs were several unusual features found in some but not all IEEs, even after doubtful IEE candidates had been eliminated (Fritzsch et al., 2016a). All IEEs, labeled by several groups, share some pathway inside the brain with FBM axons in all vertebrates (Roberts and Meredith, 1992; Fritzsch, 1999) and may even exit the brain with FBM axons to reroute to the ear in the joined facial-octaval nerve root. Only a minority of vertebrates have IEEs that remain ipsilateral: lampreys and frogs show an IEE cellular distribution that is closely associated and almost indistinguishable from FBMs (Fritzsch et al., 1989; Hellmann and Fritzsch, 1996) (Figure 1). In craniates, IEEs are bilaterally distributed (Fritzsch, 1999), a feature only shared with very few other motor neurons such as the trochlear motor neurons in lampreys (Fritzsch et al., 1990) and a subset of oculomotor motor neurons in all vertebrates investigated thus far (Fritzsch et al., 1990, 1995; Cheng et al., 2014). Another distinguishing feature of IEEs is their sometimes extremely widespread distribution of axonal branches that can cover adjacent organ systems such as multiple inner ear sensory epithelia and can be bilateral to both ears (Fritzsch and Wahnschaffe, 1987; Cowan et al., 2000) or even connect both inner ear and lateral line organs (Hellmann and Fritzsch, 1996). This highly branched and wide distribution of IEE axon bifurcations is more reminiscent of reticular neurons in the brainstem and not shared with typical motor neurons that target a given muscle. Furthermore, while IEEs are in most cases topographically closely associated with FBMs, in particular mammalian IEEs to the organ of Corti show an unusual distribution of their cell bodies associated with second order auditory nuclei of the brainstem, the superior olivary nuclei, and associated nuclei (Roberts and Meredith, 1992; Sienknecht et al., 2014). The olivo-cochlear efferents (OCEs) can be subdivided into the medial olivo-cochlear (MOC) projecting to outer hair cells and the lateral olivo-cochlear (LOC) system ending on type I afferents adjacent to inner hair cells (Simmons et al., 2011). Only MOC remain cholinergic whereas LOC fibers switch to other transmitters during development (Simmons et al., 2011).

In summary, IEEs apparently co-evolved with the vertebrate ear and are evolutionarily derived from FBMs, showing a complicated cellular distribution and axonal targets across various lineages of vertebrates.

## Chrna 7.8.9 are the phylogenetically oldest cholinergic receptor units and evolved before chordates

Obviously, if FBMs evolved in chordates one would expect that the evolution of nicotinic acetylcholine receptors (nAChR encoded by the Chrna genes) predates chordates. Indeed, the molecular origin of these receptors occurred over 1 billion years ago. Recent cladistic analysis indicates a rapid multiplication and molecular diversification into several subunits around 800 million years ago (Li et al., 2016) when multicellular animals evolved out of single celled ancestors (Figure 1). The oldest Chrna subunits, Chrna7 and 8, are found both in vertebrates and invertebrates (Papke, 2014). The two Chrna subunits now associated with the efferent innervation of the vertebrate hair cells, Chrna 9 and 10 (Elgoyhen et al., 1994, 2001; Goutman et al., 2015), show a very early split from other Chrna subunits and appear to be exclusively associated with vertebrates (Li et al., 2016). Neither Chrna 9 nor 10 have been identified in lancelet or ascidians. If present in the respective genomes, their cellular distribution could support possible homology of mechanosensory cells (Fritzsch et al., 2007) by showing expression in those sensory cells that receive efferent terminals (Burighel et al., 2011). Chrna 7, 8, 9, and 10 are recognized in lampreys but their distribution has not been verified experimentally (Smith et al., 2013). It is important to note that these ancestral Chrna units can form either pentameric homo- or hetero-multimeres with each other (Papke, 2014; Li et al., 2016) but details in the ear are not yet clear (Katz et al., 2011). Lymphocytes also express Chrna9 and 10 (Lustig et al., 2001) arguing for a function on a free floating cell that is distinct from that in a synapse. In addition, some proteins associated with motor neuron synapses have been associated with the hair cell/efferent synapse (Osman et al., 2008; Roux et al., 2016)

While the function of the IEEs in the lateral line and vestibular part of the inner ear is extensively debated (Sienknecht et al., 2014), there is agreement on a subset of IEE's function on certain hair cells of the mammalian ear, the medial OCEs ending on outer hair cells. Outer hair cells have the ability to contract to alter the tuning properties to sound stimulation (Zheng et al., 2000; Dallos et al., 2008) using a highly derived Slc channel, Prestin (Franchini and Elgoyhen, 2006; Okoruwa et al., 2008; Tan et al., 2011; Tang et al., 2013; Goutman et al., 2015). The efferent system affects the contractility of the OHC via the Chrna 9 and 10 stimulation (Elgoyhen and Franchini, 2011; Katz et al., 2011). Like Chrna 9 and 10, Prestin shows an early evolution that is not associated with its later function as the outer hair cell motor protein (Franchini and Elgoyhen, 2006) which appears to be closely associated with the evolution of the outer hair cell type only in mammals (Okoruwa et al., 2008; Tan et al., 2011; Fritzsch et al., 2013; Tang et al., 2013). It was proposed that type II afferents provide a reflex to activate MOC efferents to OHCs (Froud et al., 2015) but this suggestion was refuted by a more recent detailed paper (Maison et al., 2016).

In summary, molecular evolution of cholinergic units associated with the efferent terminals in the vertebrate inner ear and the evolution of the unique mammalian contractile protein Prestin is ancestral to the evolution of the ear. Molecular cues enabling efferents to terminate on hair cells possibly evolved before efferents engaged in forming synapses on hair cells to affect the physiology of hair cells. In fact, Chrna9 and Chrna10 are expressed on unusual cells such as lymphocytes (Lustig et al., 2001), possibly together with Chrna7 (Costantini et al., 2015), indicating that these ancestral units have a function besides those associated with synapses (Del Bufalo et al., 2014; Papke, 2014). AChR function on free floating lymphocytes may harken back to that of AChR function in single celled organisms where evolution of these receptors started clearly before multicellular organisms recruited these receptors some 800 million years ago (Figure 1) to function in cholinergic efferent terminals on mechanosensory cells that eventually evolved into inner ear and lateral line mechanosensory hair cells.

## Development of IEEs shows their origin with and segregation from FBMs through differential migration and axonal projections

**Figure 2:**
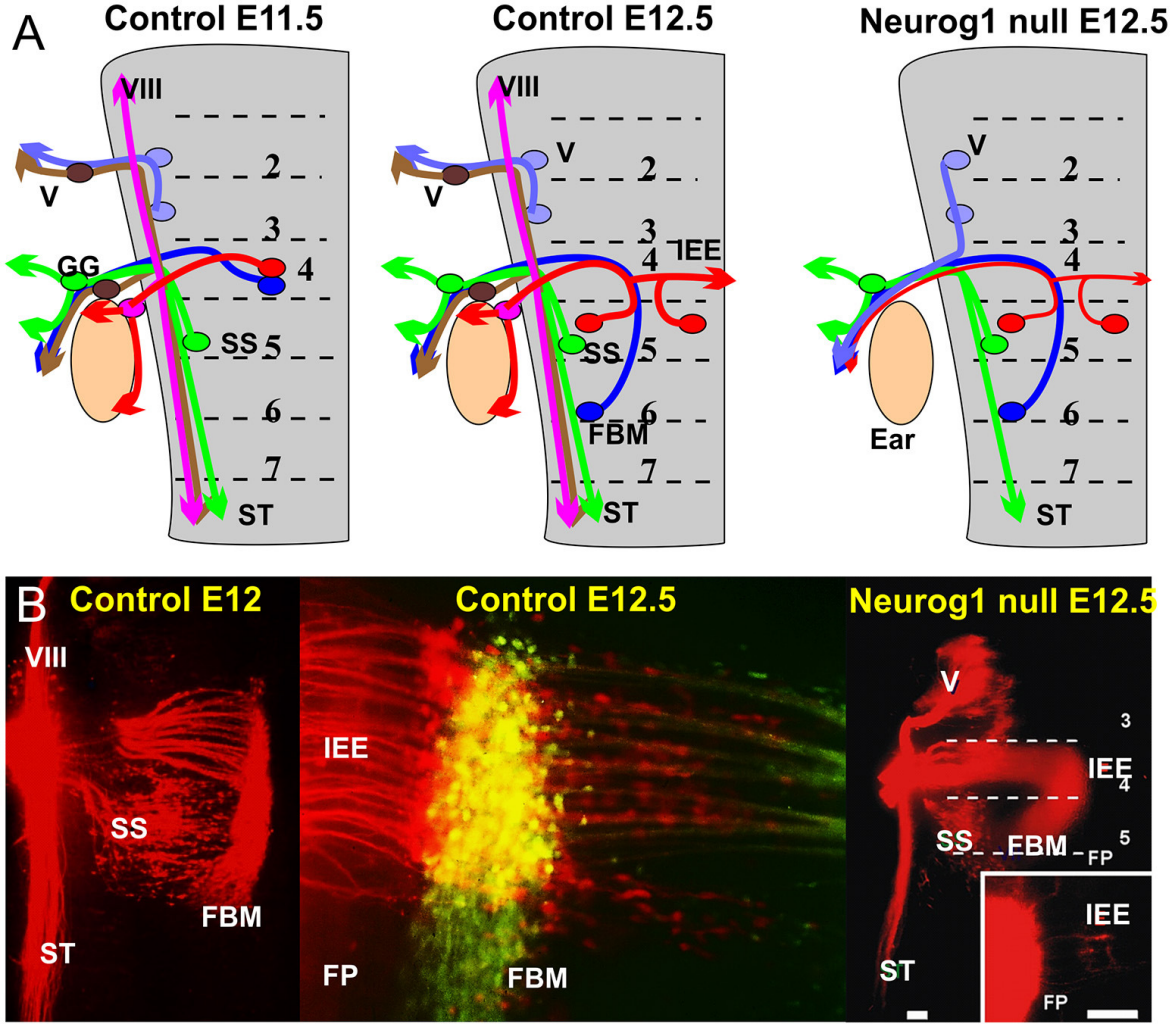
**(A)** The distribution of afferents and efferents, including the FBM and IEE is shown for a flat mounted hindbrain of an 11.5 day old mouse embryos prior to migration and a 12.5 day mouse embryo after migration. Note that the trigeminal afferents (V) and efferents enter/exit at r2, superior salvatory (parasympathetic visceral efferents) are in r5 and exit with visceral afferents running in the solitary tract (ST). FBM and IEEs in r4 and ran with afferents of the geniculate ganglion (GG) and inner ear afferents (VIII). Absence of Neurog1 eliminates all neural crest derived sensory neurons but retains the visceral sensory neurons of the GG to project to the solitary tract. Trigeminal efferents cannot exit through r2 but instead exit through r4 together with FBM and reduced numbers of IEEs. **(B)** The photomicrographs show the distribution of FBMs, SS and IEEs at E12 and the appearance of bilateral IEE overlapping with FBM at E12.5 (center). In Neurog1 mutant mice facial labeling also labels trigeminal motor neurons as well as FBM, SS and a reduced number of bilateral IEEs. Only the GG afferents forming the solitary tract remain. Bar indicates 100 um. Modified after Ma et al. (2000) and Fritzsch and Nichols (1993).

The IEEs originate from rhombomere 4 (r4; Figure 2) in all vertebrates in which their development has been investigated, and are closely associated with FBM neurons (Fritzsch and Nichols, 1993; Fritzsch et al., 1993; Bruce et al., 1997; Fritzsch, 1998a; Simmons et al., 2011). However, while FBMs can originate from the entire or only the caudal half of r4 (Figure 1) with variable addition of r3/r5 in different vertebrate lineages (Fritzsch and Nichols, 1993; Fritzsch et al., 1993; Bruce et al., 1997; Fritzsch, 1998a, 1999), IEE apparently always originate in r4 but later can disperse through differential migration to other rhombomeres (Fritzsch, 1999). It is noteworthy that FBMs are among the very few motor neurons that show longitudinal migration (Figure 1). Some motor neurons migrate in some vertebrates to the spinal cord (Fritzsch, 1998b). Migration of FBMs and IEEs depends on various components of the planar cell polarity system such as Prickle1, Vangl2, and Celsr (Qu et al., 2010; Glasco et al., 2012, 2016; Yang et al., 2014) and IEEs respond differently to these very same signals.

In addition to the variable longitudinal migration, IEEs have a variable unilateral or bilateral distribution in several vertebrate lineages (Fritzsch, 1999). The contralateral distribution is achieved either via migration across the floor plate (Fritzsch et al., 1993) or through branching of axons (Cowan et al., 2000). As a consequence, animals with contralateral migrating IEEs have few or no bilaterally projecting IEEs whereas animals with bilaterally branching IEE axons have a variable number of those cells interconnecting both ears (Fritzsch, 1999). Labeling with differently colored tracers show in all instances that IEE and FBM axons overlap in r4 and may even exit together through the facial nerve (Fritzsch et al., 1993; Simmons et al., 2011) before they segregate through differential migration/projection (Müller et al., 2003). Mutant mice bred to lack inner ear afferents through deletion of the basic Helix-Loop-Helix protein Neurog1 have several contralateral IEEs that project into the facial nerve (Figure 2), indicating that IEE preferentially grow along inner ear afferents (Ma et al., 2000) but can grow along FBM axons in the absence of an afferent ear innervation. In most vertebrates IEEs segregate at the facial nerve root from FBMs but in mammals this segregation is inside the brainstem (Simmons et al., 2011). The molecular mechanisms for the divergence from the initial FBM pathway at the facial genu inside or the facial nerve outside the brainstem remains unclear. An intriguing interaction with fibers of the dorsal acoustic stria in mice implies that IEE may segregate in mammals as an interaction with these second order auditory fibers (Gurung and Fritzsch, 2004) after the unique dorsal cochlear nucleus of mammals evolved in rhombomere 5 (Fritzsch et al., 2006; Maricich et al., 2009). Generating animals without a dorsal cochlear nucleus using targeted deletion of Ptf1a (Iskusnykh et al., 2016) could demonstrate causality.

Genes that define branchial and visceral motor neurons (Phox2b) in brainstem of mice (Pattyn et al., 1999) also eliminate IEEs, indicating that they derive from branchial motor neurons (Tiveron et al., 2003). While it is clear that IEE share the molecular basis with branchial and visceral motor neuron origin (Tiveron et al., 2003) little is known about the possible molecular basis of efferent segregation from FBM (Simmons et al., 2011). One leading candidate is the transcription factor Gata3 that shows derailed efferent projection in mutants entirely lacking Gata3 (Karis et al., 2001). It remained unclear if these effects were due to Gata3 loss in IEEs, the inner ear, or both. Subsequent conditional deletion of Gata3 in the ear show normal efferent projections to vestibular organs ruling out a direct effect of afferents (Duncan and Fritzsch, 2013) but leave the possibility open that Gata3 positive OC fibers navigate selectively along Gata3 positive afferents. Conditional deletion of Gata3 expression using motor neuron-specific cre lines such as Isl1-cre (Dvorakova et al., 2016) are needed to further evaluate the function of Gata3 in IEE pathfinding development. The bHLH gene Ascl1 also affects some aspects of navigation of IEEs (Tiveron et al., 2003).

In summary, IEE develop initially in close proximity or overlapping with FBMs, share molecular cues with FBMs, but segregate through differential projection of axons outside and inside the brain as well as differential longitudinal and radial migration, including migration and or projection of axon branches across the floor plate. Candidate transcription factors are identified that may guide the differential projection and migration of IEEs but more work is needed to support those notions.

## Experimental evidence for rerouting of spinal and ocular motor neurons to become IEEs to transplanted ears

The close proximity and initial molecular similarity of the IEEs to the FBMs (Roberts and Meredith, 1992) and their similarities during development support the hypothesis that IEEs are rerouted FBMs (Fritzsch et al., 1993; Tiveron et al., 2003). In vertebrates, the ear develops at the rostral boundary of the somites that form out of mesoderm along the notochord (Cooke, 1978; Chung et al., 1989; Huang et al., 1997). In outgroups lacking ears, lancelets (or amphioxus), somites are found extending more rostrally (Bardet et al., 2005). This implies that in craniates, the ear replaces minimally mesoderm but perhaps somites present in craniate ancestors lacking ears (Mallatt and Holland, 2013). Should this be true, then the FBMs that were destined to innervate such mesoderm or somite-derived muscle fibers may have been rerouted to innervate the ear following the evolution of the ear in ancestral craniates (Fritzsch et al., 2007). In other systems, evolution of sympathetic visceral motor neurons out of somatic motor neurons in the spinal cord and parasympathetic motor neurons out of branchial motor neurons in the brainstem (Fritzsch and Northcutt, 1993; Espinosa-Medina et al., 2016) required those neurons to access a new target that formed out of neural crest in jawed vertebrates (Fritzsch and Northcutt, 1993; Häming et al., 2011; Espinosa-Medina et al., 2014). That certain motor neurons can react plastically to reach different targets is well-known for the ocular motor system and in certain pathologies such Duane's syndrome (Fritzsch et al., 1995; Cheng et al., 2014). Moreover, addition of a limb through FGF-induction or transplantation resulted in rerouting of motor neurons destined to innervate the trunk to innervate the additional limb (Mendell and Hollyday, 1976; Turney et al., 2003).

Motor neurons can be experimentally rerouted to become IEE to the ear by transplanting ears in *Xenopus laevis* embryos caudally to the trunk to replace a somite (Elliott and Fritzsch, 2010) or rostrally into the orbit to replace the eye (Elliott et al., 2013). These two different positions show that both spinal (visceral and/or somatic) motor neurons or brainstem ocular motor neurons reroute to innervate hair cells in the transplanted ear, respectively. Ears transplanted to the trunk or orbit received “efferent” innervation from spinal or ocular motor neurons, respectively, as shown by cholinergic markers (Elliott and Fritzsch, 2010; Elliott et al., 2013) and vesicle-filled terminals (Elliott and Fritzsch, 2010).

In addition to visceral motor neurons acquiring a novel target (Espinosa-Medina et al., 2014, 2016), this is the second experimental evidence testing such evolutionary reorganization to a novel target. We speculate that ear transplantations recapitulate how evolving ears replaced one or more somites/cranial mesoderm capturing the already existing FBMs of chordates (Dufour et al., 2006) to become IEEs. Our data suggest that the ability to become IEEs is not a unique property of FBM neurons but maybe widespread among all types of motor neurons. The ear, unlike other targets of motor innervation (Elliott et al., 2013; Espinosa-Medina et al., 2016), can be innervated by any motor neuron tested thus far. This indicates possibly unique features of inner ear hair cells and may be due to the presence of the more ancestral Chrna9 and Chrna10 (Li et al., 2016) on hair cells (Elgoyhen and Franchini, 2011; Katz et al., 2011). Perhaps, then, all motor neurons retain the ability to form synapses on these receptors in all vertebrates, a hypothesis we are currently testing through ear transplantations in chickens.

Together, these data imply that the formation of IEEs may have two evolutionary and developmental significant steps:

1. The conserved nature of the cholinergic receptor associated with hair cells that closely resembles ancestral pentameric cholinergic receptors (Li et al., 2016) capable of interacting with any motor neuron terminal to form a synapse (Elliott and Fritzsch, 2010).
2. A chance event that captured already existing chordate FBM axons (Fritzsch and Northcutt, 1993; Dufour et al., 2006) simply because the inner ear evolved in the trajectory of these motor neurons out of preplacodal ectoderm through selective upregulation of Pax2/8 and other transcription factors (Bouchard et al., 2010; Riddiford and Schlosser, 2016).

It remains unclear how developmental reorganization evolved an ear out of distributed mechanosensory cells (Fritzsch et al., 2007). However, neurons that lose their target, and thus neurotrophic support released form their target, often degenerate and die during a critical period (Gould and Enomoto, 2009; Elliott et al., 2015). An alternate target, in this case the ear, would have provided neurotrophic support (Fritzsch et al., 2004; Green et al., 2012) to maintain the displaced FBMs by the very same trophic molecules that have thus far been identified to support motor neurons such as GDNF, neurturin, artemin, and CNTF (Oppenheim et al., 1995; Bailey and Green, 2014). All vertebrates have these trophic factors associated with afferent development of the ear and motor neuron maintenance (Airaksinen et al., 2006; Fritzsch et al., 2016b), but it remains to be shown when in evolution the motor neuron-specific neurotrophic factors became associated with the ear to generate a precondition to rescue rerouted motor neurons as much as we could show for the experimentally transplanted ears. How the innervation by IEEs of the ear acquired guidance cues to reach the ear remains to be shown (Karis et al., 2001; Battisti et al., 2014; Mao et al., 2014; Coate et al., 2015).

## Author contributions

BF Conceived and wrote a partial initial draft, KE completed the draft and edited.

### Conflict of interest statement

The authors declare that the research was conducted in the absence of any commercial or financial relationships that could be construed as a potential conflict of interest.
